# Evaluation of low density array technology for quantitative parallel measurement of multiple genes in human tissue

**DOI:** 10.1186/1471-2164-7-34

**Published:** 2006-02-24

**Authors:** Andrew B Goulter, Daniel W Harmer, Kenneth L Clark

**Affiliations:** 1 Pharmagene Laboratories Ltd, 2 Orchard Rd, Royston, Hertfordshire, SG8 5HD, UK

## Abstract

**Background:**

Low density arrays (LDAs) have recently been introduced as a novel approach to gene expression profiling. Based on real time quantitative RT-PCR (QRT-PCR), these arrays enable a more focused and sensitive approach to the study of gene expression than gene chips, while offering higher throughput than more established approaches to QRT-PCR. We have now evaluated LDAs as a means of determining the expression of multiple genes simultaneously in human tissues and cells.

**Results:**

Comparisons between LDAs reveal low variability, with correlation coefficients close to 1. By performing 2-fold and 10-fold serial dilutions of cDNA samples in the LDAs we determined a clear linear relationship between the gene expression data points over 5 orders of magnitude. We also showed that it is possible to use LDAs to accurately and quantitatively detect 2-fold changes in target copy number as well as measuring genes that are expressed with low and high copy numbers in the range of 1 × 10^2 ^– 1 × 10^6 ^copies. Furthermore, the data generated by the LDA from a cell based pharmacological study were comparable to data generated by conventional QRT-PCR.

**Conclusion:**

LDAs represent a valuable new approach for sensitive and quantitative gene expression profiling.

## Background

Gene expression analysis provides a view of transcriptional activity and regulation, which is useful as an indicator of likely protein expression and potential function. The current approaches to measuring gene expression vary from parallel semi-quantitative measurements of large numbers of mRNA transcripts in a single sample, using gene chip technologies, to focussed sensitive approaches, such as QRT-PCR, that enable quantitative evaluation of the expression of single genes in multiple samples [1,2]. While gene chip technology offers the means to carry out hypothesis-free investigation of the expression of a large number of genes, a major disadvantage of this technology is its lack of sensitivity. In contrast, QRT-PCR is currently capable of measuring single genes in a uniquely robust and sensitive fashion being capable of detecting specific mRNAs at levels as low as 1 copy per 5,000 cells (1 copy in 10 ng of total RNA).

For some time, there have been attempts to identify technologies that can facilitate more quantitative and sensitive expression profiling of a number of genes in single samples simultaneously. In this present study, the potential of one such technology (Low Density Array. LDA; Applied Biosystems; CA, USA) has been assessed. LDA technology is based on the QRT-PCR platform, therefore, while retaining the sensitivity of QRT-PCR, LDAs are claimed to allow the simultaneous quantification of large numbers of target genes in single samples [3]. Consequently, LDA technology could be used to compare levels of expression of panels of specific genes in treated and untreated cells *in vitro*, and also to look at the relative expression levels of panels of genes in diseased versus non-diseased tissues.

In the current study, the potential of LDAs for the evaluation of gene expression profiles in human cells and tissues has been assessed. Key features of the assessment included ease of use, sensitivity and reproducibility. In addition, to evaluate the ability of LDAs to allow the accurate measurement of changes in the expression of multiple genes, the technique was applied to thrombin-stimulated human endothelial cells, and the results compared with those generated using conventional QRT-PCR.

## Results

### Variability of gene expression data derived from LDAs

**Figure 1:**
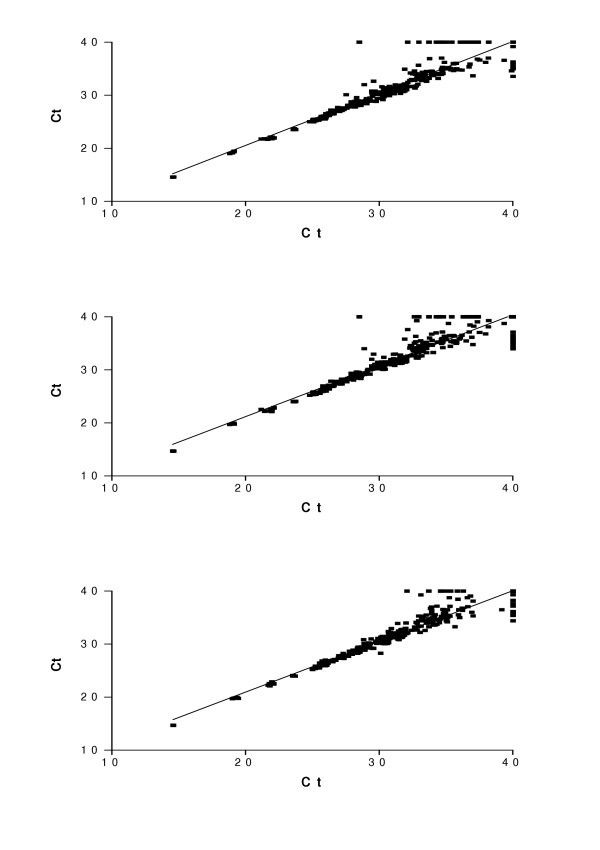
**LDAs Ct (copy threshold) data plotted LDA 1 vs 2; 1 vs 3 and 2 vs 3**. The x and y-axis represent Ct values obtained from LDA 1 and 2, LDA 1 and 3 and LDA 2 and 3 respectively. A line of best fit has been plotted.

A single sample of cDNA from human liver was run down all eight channels of three identical LDAs from the same production batch. The data derived across these LDAs were virtually identical, with correlation coefficients approximating unity (LDA 1 vs 2, 0.96. LDA 1 vs 3, 0.96. LDA 2 vs 3, 0.97). These data therefore demonstrate negligible variation between arrays from the same batch. Representative data are displayed graphically in Figure 1. Ct values (the fractional cycle number at which a PCR product is first detected) for LDA 1 have been plotted against Ct values for LDA 2. The results are more reproducible at the lower Ct values (higher copy numbers) when compared to the higher Ct values, where detection limits of the system are being approached.

The data generated for LDAs 1–3 were used to assess the quadruplicated values for each gene on each LDA, and also to look at the variation of the 12 values across the three LDAs. The data showed low coefficient of variation (CV%) between wells within a single LDA, and between LDAs. As expected, the CV% increased with lower gene expression levels i.e. higher Cts. Combined data for all targets across the three identical arrays are shown in Table 1.

### Fidelity and dynamic range of LDAs

**Figure 2:**
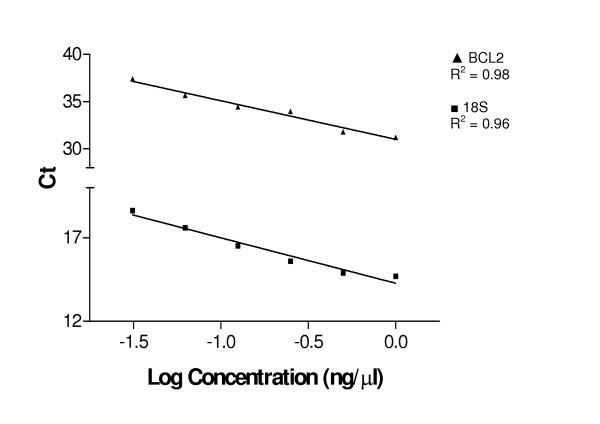
**Measurement by LDA of mRNA's for two genes (BCL2 and 18S) derived from a 2-fold dilution series of liver cDNA**. Each data point represents the mean Ct (copy threshold) value derived from quadruplicate measurement of each sample dilution. A line of best fit and R^2 ^value were derived using PRISM software.

For the liver cDNA, 2-fold dilution plots are shown (Figure 2) for two genes, 18S-ribosomal RNA (18S), which showed high expression in the liver, and BCL2, which exhibited much lower expression. These results demonstrate that these two primer probe sets generate linear results over the expression range studied, with R^2 ^values close to 1 being obtained.

These data demonstrate that using the LDAs it is possible to accurately detect 2-fold changes in target copy number. They also demonstrate that these small changes can be detected at both low Cts (high expression) and high Cts (low expression), within the approximate range of 1 × 10^2 ^– 1 × 10^6 ^copies.

**Figure 3:**
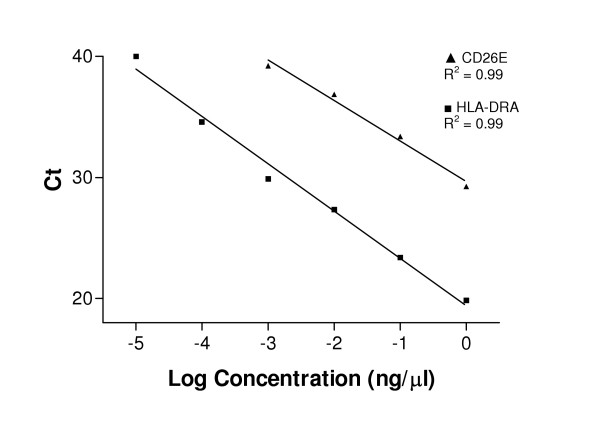
**Measurement by LDA of mRNA's for two genes (CD26E and HLA-DRA) derived from a 10-fold dilution series of tonsil cDNA**. Each data point represents the mean Ct (copy threshold) value derived from quadruplicate measurement of each sample dilution. A line of best fit and R^2 ^value were derived using PRISM software.

For the 10-fold dilution series of cDNA (derived from tonsil), the R^2^values were also determined, Figure 3, shows examples of the plots generated for two genes, HLA-DRA, which was highly expressed in the tonsil, and CD26E, which had lower expression. For CD26E, with a higher starting Ct value, it was not possible to plot all the points in the dilution series, as the sample was diluted beyond the level of detection. However, for both genes, we determined a clear linear relationship between the gene expression data points and for HLA-DRA linearity was obtained over 5 log base 10 dilutions.

### Comparing efficiencies of the 96 primer probe sets

For the liver cDNA 2-fold dilution series, R^2 ^values and slopes were determined by linear regression analysis. Data for all genes are shown in Table 2. The Ct values of the undiluted cDNA for the targets range from 14.7 to 40 with good linearity for most of the targets over the 2-fold dilution series, most with R^2 ^values close to 1. However over the range of Ct values the slopes vary, 18S (mean Ct 14.71) has a slope of -2.72 whereas BCL2 (mean Ct 31.22) has a slope of -4.08. This data indicates different amplification efficiencies of the primer probe sets.

### Comparison of gene expression profiles derived using LDAs with those derived using conventional QRT-PCR

**Figure 4:**
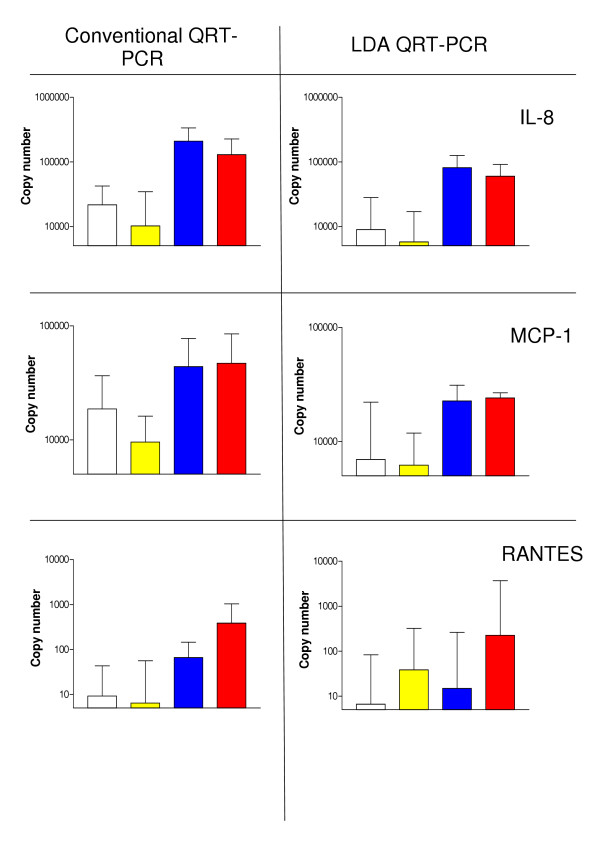
**IL-8, MCP-1 and RANTES expression in human aortic endothelial cells measured using Conventional- and LDA-QRT-PCR**. White bar; control-2 hrs. Yellow bar; control-12 hrs. Blue bar; thrombin-2 hrs. Red bar; thrombin-12 hrs. Each bar represents the geometric mean copy number (n = 3) +95 % CI.

The levels of IL-8, MCP-1 and RANTES were quantified using conventional QRT-PCR and LDA-based QRT-PCR in control and thrombin-treated human aortic endothelial cells (HAECs) at 2 and 12 hour time points (see Figure 4). Both approaches produced qualitatively and quantitatively similar data in that the expression of all three genes was up-regulated by thrombin to similar degrees. Thus, conventional QRT-PCR showed that IL-8 was up-regulated by 10-fold at 2 hours, and by 13-fold at 12 hours, while the LDA-derived data demonstrated a 9-fold increase in IL-8 at 2 hours, and 10-fold at 12 hours. MCP-1 expression data showed similar trends whether measured by either system, with 2 and 5-fold increases in the levels of transcript at 2 and 12 hours respectively, following incubation with thrombin. The level of basal RANTES expression was low, however, it was still possible using conventional and LDA-based QRT-PCR to demonstrate up-regulation in expression 12 hours post-thrombin.

**Figure 5:**
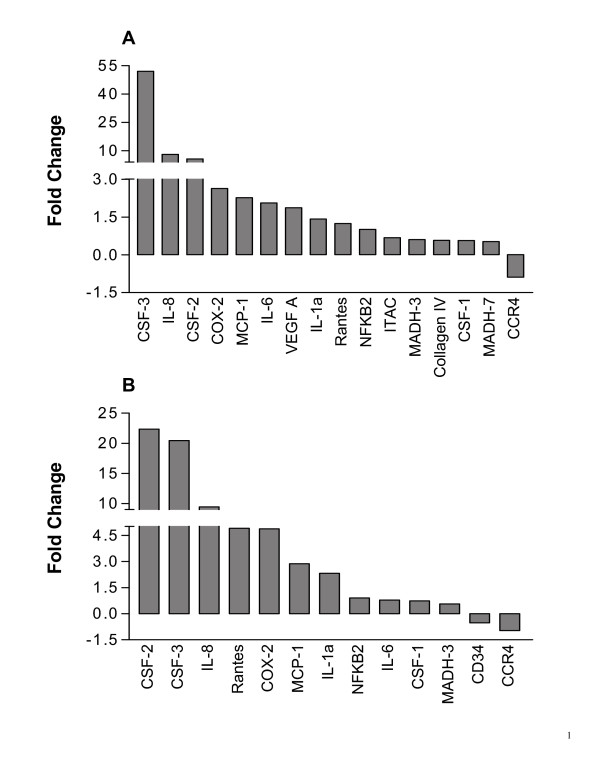
**Quantification of changes in gene expression in thrombin treated human aortic endothelial cells determined using the LDA based QRT-PCR**. Each bar represents the fold change from control (n = 3; based on geometric mean data) at 2 hours (A) and 12 hours (B) post-thrombin. Targets that were altered by less than 50 % from the control were excluded from this figure.

As the LDAs contained 96 primer probe sets in quadruplicate, it was possible to assess the effects of thrombin treatment on many more genes in a single sample than would be possible with conventional QRT-PCR. Of the 96 genes investigated, 16 at two hours and 13 at 12 hours were changed by more than 50% from the control. For all targets for which gene expression was changed by more than 50% the majority were up-regulated in response to thrombin, with only two genes being down-regulated (Figure 5).

Where target expression was compared between treatment and control groups, the expression levels of housekeeping genes (conventional QRT-PCR; β-actin, transferrin receptor and GAPDH. LDA QRT-PCR; 18S, β-actin and GAPDH), were comparable in the treated and control samples (data not shown).

## Discussion

Gene expression provides a view of transcriptional activity and regulation, and is often used as a surrogate marker for protein levels. Analysis of gene expression is used in a variety of studies, which range from identifying target genes that are altered in diseased tissues, to the screening of chemical libraries in phenotypic assays. The current approaches to measuring gene expression vary from technologies that involve parallel quantification of large numbers of mRNA transcripts in single samples (for example, using gene chip technologies) to very focussed approaches (such as QRT-PCR) that look at the expression of single genes in multiple samples. For some time, a technology has been sought that combines the ability to measure the expression of many genes in a single sample as with the chip array approach, while retaining the sensitivity and quantitative capacity offered by QRT-PCR. We have now evaluated the potential of LDA as such a technology.

The aim of the study was therefore to assess the LDA technology, and to determine whether it is capable of detecting and measuring changes in a large number of genes simultaneously in a sensitive, robust and accurate manner. Initially, we set out to investigate the variability of the technique. cDNA from one common liver sample was loaded in all channels across three arrays. Each gene was investigated in quadruplicate on each of the three arrays (all from the same batch). This facilitated investigating the variability across the quadruplicate values for each array and between triplicate arrays. The resulting data indicate that the variability between LDAs within a batch is low, with correlation coefficients for between array comparisons all being greater than 0.96. Additionally, analysis of the quadruplicate value for each gene on individual arrays revealed a low % CV, indicating a high level of reproducibility of data within each array. However, the current study has not addressed variability between cards from different batches and so the possibility exists of larger variation occurring between LDAs from different batches.

Having determined that the variability within and between the arrays was low, we next wanted to determine how capable the LDAs are of accurately quantifying small changes in gene expression. Linear regression analysis of the 2-fold serial dilution of liver cDNA across 6 LDAs showed for most genes, R^2 ^values close to 1, indicating a clear linear relationship of the gene expression data points over the range of the dilution series. The data demonstrate that LDAs are able to accurately and quantitatively detect 2-fold changes in target copy number as was demonstrated using a serial dilution of cDNA. It is hoped that the LDA cards will demonstrate the same fidelity in other experimental scenarios including those where 2 different RNA preparations are used from treated cells and untreated control cells. The data in Figures 4 and 5 support this, whereby modest changes were observed. In addition to assessing the accuracy and ability of the LDAs to detect small changes in gene expression, the linear regression analysis enabled the assessment of the slopes for each gene, with the slope indicating the efficiency of the primer probe set. The efficiencies of the primer probe sets used in this study did vary and so the delta delta Ct analysis system utilised by the ABI software for analysing the LDA data may not always be appropriate (see data analysis section) as it works on the principle that the efficiencies of the different primer probe sets are comparable.

Next, we sought to assess whether LDAs are equally capable of detecting genes that are present at low and high copy numbers. Comparison of the dynamic range of LDAs was carried out using a 10-fold serial dilution of cDNA prepared from tonsil. The same channels (same genes) of 6 separate LDA cards were loaded with six concentrations of tonsil cDNA. For targets with a copy threshold of less than 40 (within the limits of detection) across the concentration range, a dynamic range of the system over 5 orders of magnitude was demonstrated, indicating that it is possible to accurately and quantitatively detect genes at high and low copy numbers. This data is in agreement with a recent study using LDA cards, which found the technology had a wide dynamic range, up to seven orders of magnitude [3].

Having built confidence regarding the sensitivity, reproducibility and dynamic range of LDAs, the next step was to apply them to an experimental situation and compare the data with those generated using conventional QRT-PCR. Thus, both techniques were used to evaluate thrombin-induced changes in gene expression in human aortic endothelial cells. The study focused on three genes, IL-8, MCP-1 and RANTES, which are implicated in atherosclerosis [4-6]. The resulting data indicate that LDAs produced qualitatively and quantitatively similar data to conventional QRT-PCR. Both techniques indicated that thrombin caused up-regulation in the three key genes of interest (IL-8, MCP-1, RANTES). This suggests that LDAs are suitable for examining changes in gene expression in response to pharmacological stimuli.

A key advantage of LDAs over conventional QRT-PCR is their ability to assess changes in multiple genes in a single sample. Therefore, in the current study in thrombin-treated cells, in addition to demonstrating that IL-8, MCP-1 and RANTES are up-regulated, the LDAs provided data on a further 93 genes. Of these genes, the expression levels of 16 at 2 hours and 13 at 12 hours were changed by more than 50% from the control. From the genes whose expression was changed by more than 50%, the majority were up-regulated in response to thrombin, with only two genes being down-regulated. These additional data demonstrate the power of the LDA technology to investigate changes in expression of a large number of genes simultaneously in a single study in a sensitive and quantitative manner. In the present study, in addition to demonstrating the expected thrombin-induced increase in IL-8 and MCP-1 mRNA, the LDAs detected for the first time that thrombin regulates several other genes (CSF-2, CSF-3, MADH-3 and MADH-7) in human endothelial cells.

Other considerations that are also important in assessing this new technology are its ease of use and cost implications. Loading and setting up the LDA cards was straightforward, with no requirement for robotic liquid handling. The cost of conducting studies on the LDAs is less than that of conventional QRT-PCR. This primarily reflects the savings made in time and money through not having to design and purchase specific primer probe sets. Additionally, LDAs require a very low reaction volume, which means that there are large savings in master mix, and a reduction in the use of RNA per study. In the current study involving 20 LDAs, we calculated the savings relative to carrying out the study using conventional QRT-PCR to be in the order of 60% on time and 70% on consumable cost.

## Conclusion

In conclusion, this study has shown that LDA technology provides a sensitive and reproducible approach to study gene expression. In addition, this new technique has been shown to produce comparable results to those produced by conventional QRT-PCR. However, LDAs have the advantage of allowing multiple genes to be studied from a single sample, and also offer savings in terms of costs and materials. Therefore, LDAs represent a valuable new approach to sensitive and quantitative gene expression profiling.

## Methods

The human tissues used in this study were obtained through partnership with medical intermediaries, hospitals and tissue banks, in all cases with the full informed consent of the donor or their next of kin, and with approval from the appropriate ethics committees. RNA was extracted from tissue using TriZol (Invitrogen Life technologies, Paisley, UK), a commercially available mixture of organic solvents according to the manufacturer's protocol.

For primary cell studies, human aortic endothelial cells (Cambrex, Nottingham, UK) (passage 4) maintained in EGM-2 (Cambrex) were plated out in collagen-coated 96-well plates, and allowed to reach approximately 80-90 % confluence (6 × 10^4 ^cells/well). Eighteen hours before commencement of the thrombin treatment, the culture medium was removed and replaced with basal medium, EBM-2 (Cambrex,) containing 2 % FBS. At time point zero, the cells were incubated with thrombin (6 Units/ml, in EBM-2), or media. The cells were incubated for 2 and 12 hours at 37°C; at 5% humidity; in 95% O_2_; 5% CO_2_. After the incubation period, the medium was removed from the cells, and the total RNA was extracted immediately using RNeasy extraction kits (Qiagen, West Sussex, UK) according to the manufacturer's protocol. To ensure the quality of the data, RNA was extracted from an equal number of cells. Each treatment was performed in triplicate and pooled. For the LDA cards the RNA samples loaded were derived from 3 donors. The expression levels of the housekeeping genes, β-actin, transferrin receptor and GAPDH, were measured in each sample using conventional QRT-PCR (data not shown). The levels of expression of the house keeping genes across the samples were not significantly different (student's paired t test on the log_10 _transformed data). Furthermore, equivalent expression as confirmed by the equivalence test [7] for dependent samples was identified for β-actin, transferrin receptor and GAPDH. Hence, normalisation of the data against a single housekeeping gene was not carried out.

Quantification of specific mRNAs by conventional QRT-PCR was carried out using previously described methodology [8]. Total RNA samples were treated with RNase-free DNase I (amplification grade; Invitrogen Life Technologies) according to the manufacturers' instructions. PCR was then carried out in absence of reverse transcriptase to ensure the removal of all genomic DNA. For each RNA sample, total RNA was then used as template for first strand cDNA synthesis. The RNA in a volume of 4 μl and in the presence of reverse primers for IL8, MCP1, RANTES and GAPDH, 1 × PCR buffer II (Applied Biosystems), and 5 mM MgCl_2 _was heated to 72°C for 5 min and cooled slowly to 55°C. After addition of all other reagents, the 6 μl reaction was incubated at 37°C for 30 min followed by an enzyme inactivation step of 90°C for 5 min. The final reaction conditions for reverse transcription were as follows: 1 × PCR buffer II; 5 mM MgCl_2_; 1 M dATP, dGTP, and dCTP, 2 mM dTTP; 12.5 U MuLV reverse transcriptase (Invitrogen Life Technologies). The resulting cDNA was subjected to PCR amplification in the ABI 7700 or 7900 Sequence Detection System to identify either IL8, MCP1 or RANTES PCR product in conjunction with GAPDH transcripts in a single reaction, appropriate positive and negative PCR controls were also included. Final reaction conditions were 4% glycerol; 0.66 × TaqMan buffer A (Applied Biosystems); 6 mM MgCl_2_; 358 μM dATP, dUTP, dGTP, and dCTP; 2.5 U AmpliTaq Gold; and 0.16 U of AmpErase UNG (uracil *N*-glycosylase). An initial enzyme activation step of 94°C for 12 min was followed by 40 PCR cycles each of 95°C for 15 s, 60°C for 30 s. Forward and reverse primers and flurogenic probes were designed to the targets of interest using Primer Express software (Applied Biosystems). All the probes were quenched with carboxytetramethyl rhodamine, and labelled with the fluor, 6-carboxyfluorescein. The primer probe sequences used in conventional QRT-PCR were designed using Primer Express. They were homology searched against all known sequences. The efficiency of the primer probe sets conformed to the global standard curve described by Bowen et al [8]. Additionally the PCR products for all primer probe sets were run down a 4% agarose gel and for all of the sets investigated a single band of the expected molecular weight was observed.

IL-8, Accession number NM_000584.

Forward 5' CTGGCCGTGGCTCTCTTG 3'

Reverse 5' CCTTGGCAAAACTGCACCTT 3'

Probe 5' CAGCCTTCCTGATTTCTGCAGCTCTGTGT 3'

MCP-1 Accession number NM_002982.

Forward 5' CGCCTCCAGCATGAAAGTCT 3'

Reverse 5' GGAATGAAGGTGGCTGCTATG 3'

Probe 5' TGCCGCCCTTCTGTGCCTGC 3'

RANTES Accession number NM_002985.

Forward 5' TGCATCTGCCTCCCCATATT 3'

Reverse 5' AGTGGGCGGGCAATGTAG 3'

Probe 5' CTCGGACACCACACCCTGCTGCT 3'

For quantification of mRNAs by LDA, cDNA was synthesised from RNA obtained from liver, tonsil and thrombin-treated aortic endothelial cells using a high capacity cDNA archive kit (Applied Biosystems, Product Number 4322171) according to the manufacturer's protocols. To assess the fidelity of LDAs to quantitatively detect small changes in copy number, cDNA from liver was then diluted to make a 2-fold dilution series, so the concentrations per well on the LDAs were 1, 0.5, 0.25, 0.125, 0.0625 and 0.03125 ng/μl. To assess dynamic range, cDNA from tonsil underwent a 10-fold dilution series, so the concentrations used were 1, 0.1, 0.01, 0.001, 0.0001 and 0.00001 ng/μl. For the primary cell studies, the concentration of cDNA used was 0.08 ng/μl for both conventional and LDA QRT-PCR.

LDA technology allows the simultaneous measurement of expression of up to 384 genes in a single sample. Each array has eight channels, allowing between one to eight cDNA samples to be loaded. Each channel services 48 wells, with the possibility of having all 48 wells containing different primer probe sets. For this study the Low Density Immune Profiling Array was used; Product number 4342510. In these arrays, each channel, with 48 wells, contains pre-designed primer probe sets for 12 different genes (in quadruplicate). Therefore, each array consists of 96 pre-designed TaqMan gene expression assays in quadruplicate. The targets include cytokines, chemokines, growth factors, immune regulators, apoptosis markers, ischemia markers, tissue-specific markers, as well as housekeeping genes. To run the arrays, cDNA is added to the PCR master mix and the resulting mixture is then added to one or all of the eight channels on the array. The samples are loaded, by centrifugation into the wells that contain the lyophilised primer probe sets. The wells are then sealed, and the array is run on the ABI 7900HT. All of these steps were carried out according to the manufacturer's protocol.

### Data analysis

The starting mRNA copy number (Cn) of a target sequence for conventional and LDA QRT-PCR is established by determining the fractional PCR threshold cycle (Ct) number at which the fluorescent signal generated during the replication process exceeds a threshold value. The initial amount of target mRNA in each sample is then estimated from the experimental Ct by extrapolation from a global standard curve generated using known amounts of genomic DNA [8].

Linear regression data analysis for 2 and 10-fold dilutions were determined using PRISM (Graphpad Software Inc., CA, USA).

## Authors' contributions

ABG, DWH and KLC participated in hypothesis design, and writing of the manuscript. ABG and DWH designed and carried out the experiments. All authors read and approved the final manuscript.

## Figures and Tables

**Table 1:** Analysis of the gene expression values, for LDA 1–3

**Gene**	**Ct Mean**	**CV%**	**Gene**	**Ct Mean**	**CV%**
18S-4342379-18S	14.62	0.44	TNF-a-Hs00174128_m1	31.66	1.42
C3-Hs00163811_m1	19.37	1.96	ITAC-Hs00171138_m1	32.05	1.63
FN precursor-Hs00365052_m1	21.87	1.75	CD62E (SELP)-Hs00174583_m1	32.09	1
GAPDH-4342376-GAPDH	22.25	1.59	ACE-Hs00174179_m1	32.1	4.55
Stat3-Hs00234174_m1	23.76	0.88	CD25-Hs00166229_m1	32.57	1.27
HLA-DRA-Hs00219575_m1	25.2	0.86	CCR5-Hs00152917_m1	33.1	2.14
CD54 (ICAM)-Hs00164932_m1	25.47	1.05	IL-6-Hs00174131_m1	33.56	3.15
HO-1-Hs00157965_m1	25.8	0.84	IL-18-Hs00155517_m1	33.78	1.96
GUSB-Hs99999908_m1	25.98	0.6	CCR4-NM_005508	33.78	5.99
CD68-Hs00154355_m1	26.08	0.88	COX-2-Hs00153133_m1	33.79	1.7
VEGF A-Hs00173626_m1	26.24	1.47	FasL-Hs00181225_m1	33.83	2.83
TGF-b-Hs00171257_m1	26.32	1.59	MEGALIN-Hs00189742_m1	33.87	3.01
MCP-1-Hs00234140_m1	26.68	1.02	CD154(TNFSF5)-Hs00163934_m1	33.96	2.01
NFKB2-Hs00174517_m1	27.2	1.1	CD34-Hs00156373_m1	34.31	2.68
BAX-Hs00180269_m1	27.21	1.05	MADH-3-Hs00232219_m1	34.66	6.11
ECE-1-Hs00154837_m1	27.31	1.77	AT1R(AGTR1)-Hs00241341_m1	34.91	3.29
Mip-1a-Hs00234142_m1	27.55	0.95	CD28-Hs00174796_m1	34.95	3.63
CYP1A2-Hs00167927_m1	27.56	1.03	Collagen IV(COL4A5)-Hs00166712_m1	35	2.2
IkB2-Hs00395088_m1	28	0.77	T-bet (TBX21)-Hs00203436_m1	35.73	4.16
IL-1b-Hs00174097_m1	28.23	2.32	CYP7A1-Hs00167982_m1	35.83	7.25
CD45-Hs00365634_g1	28.25	1.12	GZMB-Hs00188051_m1	35.86	3.05
CD38-Hs00233552_m1	28.33	0.7	TNF-b-Hs00236874_m1	36.34	10.21
GNLY-Hs00246266_m1	28.38	1.65	IFN-g-Hs00174143_m1	36.69	7.65
CSF-1-Hs00174164_m1	28.49	1.18	CD19-Hs00174333_m1	37.32	6.42
CD40-Hs00374176_m1	28.71	0.93	CD152-Hs00175480_m1	37.33	7.63
HLADR-NM_002124	28.77	1.01	IL-15-Hs00174106_m1	37.51	2.79
Rantes-Hs00174575_m1	28.82	1.11	IL-12p35-Hs00168405_m1	37.55	4.59
Mip-3c-Hs00171149_m1	28.88	1.64	CCR7-Hs00171054_m1	37.8	4.16
Fas-Hs00163653_m1	28.99	2.07	ICOS-Hs00359999_m1	37.96	5.78
CD71-Hs99999911_m1	29.02	1.04	CXCR3-Hs00171041_m1	38.23	5.79
IL-7-Hs00174202_m1	29.51	1.84	IL-1a-Hs00174092_m1	38.9	3.39
SKI-Hs00161707_m1	29.63	2.05	IL-3-Hs00174117_m1	39	6
MADH-7-Hs00178696_m1	30.19	1.2	IL-5-Hs00174200_m1	39.08	5.75
Beta Actin-Hs00174609_m1	30.27	1.48	IL-17-Hs00174383_m1	39.22	4.63
PRF1-Hs00169473_m1	30.43	1.24	iNos(Nos2A)-Hs00167248_m1	39.24	4.54
BCL-XL-Hs00169141_m1	30.49	1.75	IL-2-Hs00174114_m1	39.26	4.46
CD8-Hs00233520_m1	30.59	1.3	IL-12p40-Hs00233688_m1	39.45	3.28
CD4-Hs00181217_m1	30.6	2.25	IL-9-Hs00174125_m1	39.73	2.32
IL-8-Hs00174103_m1	30.69	3.06	RENIN(REN)-Hs00166915_m1	39.78	1.94
BCL2-Hs00153350_m1	30.84	1.17	AT2R(AGTR2)-Hs00169126_m1	40	0
CD3-Hs00167894_m1	31.06	1.8	CCR2-Hs00174150_m1	40	0
IL-10-Hs00174086_m1	31.16	1.29	CSF-2-Hs00171266_m1	40	0
CD62E (SELE)-Hs00174057_m1	31.33	1.29	CSF-3-Hs00357085_g1	40	0
IP10-Hs00171042_m1	31.42	1.22	GITR(TNFRSF18)-Hs00188346_m1	40	0
CD86-Hs00199349_m1	31.42	1.59	IL-13-Hs00174379_m1	40	0
Endothelin(EDN1)-Hs00174961_m1	31.48	1.26	IL-4-Hs00174122_m1	40	0
CD80-Hs00175478_m1	31.49	1.81	MYH6-Hs00411908_m1	40	0

This table shows the mean data (from liver cDNA) generated from the 94 primer probe sets across the three arrays of the same format. There were quadruplicate determinations for each gene on each card so the table represents 12 measurements for each gene. Mean Ct (copy threshold) and coefficient of variation (CV%) have been calculated.

**Table 2:** Linear regression analysis of the 2-fold dilution data

**Gene**	**Mean Ct**	**Slope**	**R^2^**	**Gene**	**Mean Ct**	**Slope**	**R^2^**
18S-4342379-18S	14.71	-2.72	0.96	CD62E (SELE)-Hs00174057_m1	31.55	-6.53	0.93
C3-Hs00163811_m1	19.83	-3.92	0.97	IL-8-Hs00174103_m1	31.55	-5.83	0.87
FN precursor-Hs00365052_m1	22.29	-3.85	0.97	IP10-Hs00171042_m1	31.75	-4.24	0.94
GAPDH-4342376-GAPDH	22.7	-3.55	0.98	Endothelin(EDN1)-Hs00174961_m1	31.77	-5.45	0.82
Stat3-Hs00234174_m1	24.03	-3.7	0.98	CD86-Hs00199349_m1	31.78	-5.3	0.93
HLA-DRA-Hs00219575_m1	25.37	-3.87	0.99	CD80-Hs00175478_m1	31.81	-5.14	0.86
CD54 (ICAM)-Hs00164932_m1	25.75	-3.74	0.99	TNF-a-Hs00174128_m1	31.84	-4.15	0.92
HO-1-Hs00157965_m1	26	-3.71	0.99	CD62E (SELP)-Hs00174583_m1	32.41	-5.07	0.96
GUSB-Hs99999908_m1	26.09	-3.67	0.97	CD25-Hs00166229_m1	32.44	-5.2	0.94
CD68-Hs00154355_m1	26.36	-4.02	0.99	ITAC-Hs00171138_m1	32.61	-5.3	0.94
VEGF A-Hs00173626_m1	26.63	-3.88	0.98	ACE-Hs00174179_m1	33.1	-4.33	0.82
TGF-b-Hs00171257_m1	26.78	-3.82	0.95	CCR5-Hs00152917_m1	33.77	-4.76	0.94
MCP-1-Hs00234140_m1	26.99	-3.85	0.98	IL-18-Hs00155517_m1	33.98	-4.25	0.88
BAX-Hs00180269_m1	27.51	-4.7	0.98	MEGALIN-Hs00189742_m1	34.1	-2.98	0.55
NFKB2-Hs00174517_m1	27.53	-3.87	0.98	COX-2-Hs00153133_m1	34.3	-3.35	0.86
CYP1A2-Hs00167927_m1	27.81	-3.71	0.99	CD154(TNFSF5)-Hs00163934_m1	34.57	-3.16	0.73
ECE-1-Hs00154837_m1	27.81	-3.81	0.97	IL-6-Hs00174131_m1	34.59	-4.09	0.93
Mip-1a-Hs00234142_m1	27.88	-3.74	0.99	FasL-Hs00181225_m1	34.74	-4	0.9
IkB2-Hs00395088_m1	28.24	-5.23	0.91	CCR4-NM_005508	34.99	-3.49	0.96
IL-1b-Hs00174097_m1	28.33	-5.27	0.9	CD34-Hs00156373_m1	35.3	-3.01	0.88
CD38-Hs00233552_m1	28.5	-3.41	0.98	CD28-Hs00174796_m1	35.53	-2.41	0.8
CD45-Hs00365634_g1	28.63	-4.16	0.98	AT1R(AGTR1)-Hs00241341_m1	35.53	-3.28	0.83
GNLY-Hs00246266_m1	28.74	-3.5	0.89	Collagen IV(COL4A5)-Hs00166712_m1	35.71	-3.2	0.82
CSF-1-Hs00174164_m1	28.86	-4.09	0.98	T-bet (TBX21)-Hs00203436_m1	35.75	-3.14	0.87
CD40-Hs00374176_m1	29.01	-3.49	0.98	CD19-Hs00174333_m1	36.35	-1.78	0.71
HLADR-NM_002124	29.11	-4.32	0.97	IL-12p35-Hs00168405_m1	36.57	-2.48	0.88
CD71-Hs99999911_m1	29.19	-3.7	0.98	IFN-g-Hs00174143_m1	36.84	-2.12	0.88
Rantes-Hs00174575_m1	29.19	-4.52	0.97	GZMB-Hs00188051_m1	36.99	-1.83	0.77
Mip-3c-Hs00171149_m1	29.43	-4.57	0.98	MADH-3-Hs00232219_m1	37.1	-2.21	0.79
Fas-Hs00163653_m1	29.5	-5.89	0.87	CYP7A1-Hs00167982_m1	37.17	-2.48	0.63
IL-7-Hs00174202_m1	29.74	-6.84	0.9	CD152-Hs00175480_m1	37.28	-1.46	0.64
SKI-Hs00161707_m1	30.31	-3.89	0.96	CXCR3-Hs00171041_m1	37.62	-1.24	0.49
MADH-7-Hs00178696_m1	30.48	-5.13	0.93	IL-15-Hs00174106_m1	37.92	-1.38	0.77
Beta Actin-Hs00174609_m1	30.57	-4.52	0.92	CCR7-Hs00171054_m1	38.15	-1.34	0.85
CD8-Hs00233520_m1	30.68	-4.61	0.99	iNos(Nos2A)-Hs00167248_m1	38.86	-0.54	0.43
PRF1-Hs00169473_m1	30.73	-4.22	0.9	IL-17-Hs00174383_m1	38.92	-0.51	0.43
BCL-XL-Hs00169141_m1	30.78	-3.37	0.99	TNF-b-Hs00236874_m1	38.97	-0.8	0.12
BCL2-Hs00153350_m1	31.22	-4.08	0.98	ICOS-Hs00359999_m1	38.98	-0.9	0.81
IL-10-Hs00174086_m1	31.33	-5.37	0.97	IL-5-Hs00174200_m1	38.98	-0.99	0.24
CD4-Hs00181217_m1	31.35	-4.93	0.97	IL-1a-Hs00174092_m1	39.39	-0.51	0.78
CD3-Hs00167894_m1	31.42	-5.68	0.93				

This table shows data (from liver cDNA) generated from the 96 primer probe sets. There were quadruplicate determinations for each sample dilution. Mean Ct (copy threshold) of the undiluted liver cDNA is given for each gene. The slope and R^2 ^values have been calculated. Genes with Ct values of 40 have not been included in the table.
